# Isotopic reconstruction of the weaning process in the archaeological population of Canímar Abajo, Cuba: A Bayesian probability mixing model approach

**DOI:** 10.1371/journal.pone.0176065

**Published:** 2017-05-01

**Authors:** Yadira Chinique de Armas, Mirjana Roksandic, Dejana Nikitović, Roberto Rodríguez Suárez, David Smith, Nadine Kanik, Dailys García Jordá, William M. Buhay

**Affiliations:** 1 Department of Anthropology, University of Winnipeg, Winnipeg, Manitoba, Canada; 2 Department of Anthropology, University of Toronto Scarborough, Toronto, Ontario, Canada; 3 Department of Anthropology, University of Toronto, Toronto, Ontario, Canada; 4 Anthropological Montane Museum, University of Havana, Havana, Cuba; 5 Department of Anthropology, University of Toronto Mississauga, Mississauga, Ontario, Canada; 6 Department of Geography, University of Winnipeg, Winnipeg, Manitoba, Canada; 7 Alberta Health Services, Calgary, Alberta, Canada; Indiana University Bloomington, UNITED STATES

## Abstract

The general lack of well-preserved juvenile skeletal remains from Caribbean archaeological sites has, in the past, prevented evaluations of juvenile dietary changes. Canímar Abajo (Cuba), with a large number of well-preserved juvenile and adult skeletal remains, provided a unique opportunity to fully assess juvenile paleodiets from an ancient Caribbean population. Ages for the start and the end of weaning and possible food sources used for weaning were inferred by combining the results of two Bayesian probability models that help to reduce some of the uncertainties inherent to bone collagen isotope based paleodiet reconstructions. Bone collagen (31 juveniles, 18 adult females) was used for carbon and nitrogen isotope analyses. The isotope results were assessed using two Bayesian probability models: Weaning Ages Reconstruction with Nitrogen isotopes and Stable Isotope Analyses in R. Breast milk seems to have been the most important protein source until two years of age with some supplementary food such as tropical fruits and root cultigens likely introduced earlier. After two, juvenile diets were likely continuously supplemented by starch rich foods such as root cultigens and legumes. By the age of three, the model results suggest that the weaning process was completed. Additional indications suggest that animal marine/riverine protein and maize, while part of the Canímar Abajo female diets, were likely not used to supplement juvenile diets. The combined use of both models here provided a more complete assessment of the weaning process for an ancient Caribbean population, indicating not only the start and end ages of weaning but also the relative importance of different food sources for different age juveniles.

## Introduction

Paleodietary studies in the insular Caribbean have traditionally focused on adults [1–3], while paleonutritional assessments of juveniles have been rarely considered, or analyzed separately from adults [4–5]. This is primarily due to the fact that very few Caribbean archaeological sites have enough well-preserved juveniles to enable proper assessments of culturally significant juvenile-specific nutritional issues such as breastfeeding and weaning. Breastfeeding and the timing of weaning vary between cultures, and are shaped by complex interactions of beliefs about health and nutrition, construction of childhood and parental identities, religious values and lifestyle [6–9]. Additionally, infant feeding practices have important implications for population dynamics because they affect fertility, morbidity and mortality patterns [6, 10–11].

Age related bone collagen carbon and nitrogen isotope changes in juvenile paleodiets have previously been used to estimate weaning ages in a variety of archaeological contexts [12–16]. Estimating weaning ages using carbon and nitrogen bone collagen isotopic compositions is challenging and typically involves uncertainties related to collagen to food source isotope fractionations, variable food type protein contents and isotopic compositions [17–18]. Furthermore differential bone turnover rates in juveniles have generally not been considered, affecting the reliability of weaning processes length interpretations [19]. Recently, a number of Bayesian probability models have been developed which permit the use of collagen-to-food source specific isotope fractionations and food source protein contents and isotopic compositions, advancements that can serve to enhance the accuracy of paleodiet reconstructions [20–24]. Additionally, another recent Bayesian probability model exclusively uses nitrogen isotopic composition differences between females and juveniles to estimate both the start and end ages of weaning. Importantly, it accounts for changing age related bone collagen turnover rates [19].

Numerous well-preserved juvenile remains from the site of Canímar Abajo (Matanzas, Cuba) provide a unique opportunity to evaluate the juvenile weaning practices of a pre-contact, insular Caribbean population of fisher-gatherers/horticulturalists. Buhay and colleagues [5] previously identified significant dietary differences between juveniles and adults from Canímar Abajo, which they suggested was evidence of early weaning. In this study, we examine the timing of the weaning process in a Canímar Abajo population and the type of sources they used as weaning foods. These goals were accomplish by using two Bayesian mixing models: Weaning Ages Reconstruction with Nitrogen isotopes (WARN) [19] and Stable Isotope Analyses in R (SIAR) [20, 25–27]. This study also discusses the value of combining WARN and SIAR model inferences, within a probabilistic framework, to obtain more robust results in weaning practice reconstructions.

### Weaning: Definition of terms

In the anthropological literature, ‘weaning’ represents both the introduction of complementary food while gradually reducing breast milk consumption, as well as, cessation of breastfeeding (also called complete weaning) [12, 28–29]. In this paper, we refer to weaning as a process that starts when foods other than breast milk are continuously included in the diet, and ends with the cessation of breastfeeding [28, 30]. Here, ‘weaning foods’ include supplementary (foods other than breast milk, consumed during the weaning process) and ‘transitional foods’ (food types which are isotopically distinct from adult food and are included in juvenile diets after the cessation of breastfeeding) while ‘adult food’ refers to the food typically consumed by adults in the population [19, 28, 30]. Please note that in this paper, the term ‘juvenile’ refers to the individuals that still undergo growth and development (from birth until the end of adolescence).

## Materials and methods

### Canímar Abajo site

**Fig 1:**
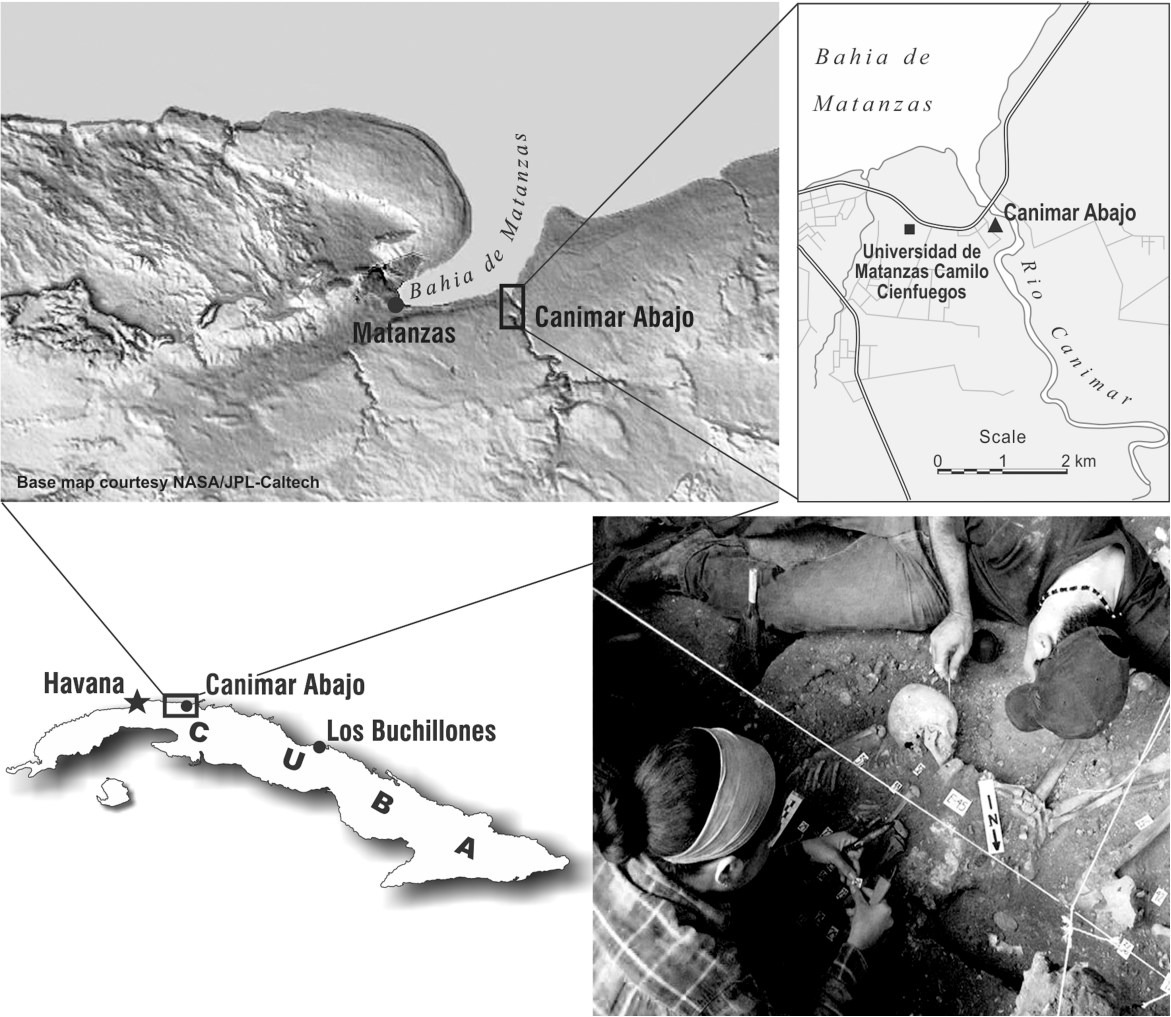
Location of Canímar Abajo, Cuba (23°2'16.29"N and 81°29'48.25"W). See image use policy in S1 File.

Canímar Abajo is a shell-matrix site located near Matanzas City (Cuba) at the estuary on the western bank of the Canímar River at 23°2'16.29"N and 81°29'48.25"W (Fig 1). The Canímar River is navigable from the Bay of Matanzas to over 11 km, reaching 12 m in depth in some areas. The estuary is a diverse ecosystem supporting both mangrove and evergreen forests [31]. The site consists of five stratigraphic levels [32] and includes two cemeteries: the Old Cemetery dated to ca.1130±110 BCE and the Young Cemetery dated to ca. 580±120 CE, separated by an approximately 1 m thick shell-midden layer [33]. Since the site was first discovered in the early 1980s, at least 213 individuals have been excavated from the two cemeteries including 130 juveniles [34]. While Canímar Abajo was first described as an exclusively fisher-gatherer population, more recent paleodietary studies showed that the use of terrestrial resources, including cultigens, played an important role in their diet [5, 35].

Permits for excavations were issued by “Comisión Nacional de Monumentos” in Cuba to Dr. Roberto Rodríguez Suárez, as project director, from 2004 to 2014 (Permits #: PEA-2/14). These permits allowed our team to excavate and study the material from Canímar Abajo, Cuba. The archaeological specimens analyzed in this study are temporary deposited at Museo Antropológico Montané, Faculty of Biology, University of Havana in Cuba and they are not publicly deposited or accessible by others in a permanent repository.

### Skeletal samples

Rib samples from 31 juveniles (Old Cemetery: 2; Young Cemetery: 29) and femur samples from 18 adult females (Old Cemetery: 5; Young Cemetery: 13) were processed for bone collagen carbon (δ^13^C) and nitrogen (δ^15^N) isotope analyses (Table 1). Ribs have a high turnover rate which makes them good candidates for reflecting dietary changes during the weaning process. Some female and juvenile isotopic values had been previously reported by us in a general Paleodietary study of the Canímar Abajo population [5]. Individuals from both cemeteries were analyzed together because no statistically significant (isotope based) dietary differences between the adults of the two cemeteries were detected [5, 35]. Furthermore, the Old Cemetery juvenile sample carbon and nitrogen isotopic compositions, while substantially smaller than the Young Cemetery sample set, still plotted within the range of values for the Young Cemetery juveniles, suggesting that combining the data sets does not influence the results.

**Table 1:** δ^13^C_col_ (‰VPDB), δ^15^N, (‰ Air), %C, %N, C/N and %Col yield values for Canímar Abajo juveniles and adult females.

Groups	Sample ID/Cemetery	Age estimates	δ^13^C_col_ (‰)	δ^15^N (‰)	%C	%N	C/N	% Col yield
**Group 1** (0–0.5 years) N = 11	E-29/YC	Birth-1.5 months	-18.12	9.45	29.60	10.60	2.80	5.60
	E-30/YC	1.5–3 months	-15.03	12.07	38.20	10.90	3.50	11.20
	E-32/YC	1.5–3 months	-16.96	9.13	30.70	9.60	3.20	7.30
	E-47/YC	Perinatal	-19.89	11.81	34.20	10.10	3.40	10.70
	E-48/YC	Birth-1.5 months	-16.70	13.70	36.30	10.70	3.40	10.50
	E-49/YC	1.5–3 months	-19.76	6.98	36.10	10.60	3.40	10.30
	E-50/YC	1.5–3 months	-21.23	9.18	36.10	10.60	3.40	11.30
	E-55/OC	1.5–3months	-20.40	11.30	36.50	10.70	3.40	6.50
	CCC (5)/YC	Birth-1.5 months	-19.01	12.53	35.90	10.90	3.30	10.20
	CCC (10)/YC	1.5–3 months	-21.60	8.29	36.20	10.60	3.40	9.30
	C (291)/YC	1.5–3 months	-17.57	14.48	36.00	11.30	3.20	2.70
**Mean** **(SD)**			-18.75 (2.06)	10.81 (2.37)	35.10 (2.50)	10.60 (0.44)	3.30 (0.20)	8.70 (2.70)
**Min/Max**			-21.60/-15.03	6.98/14.48	29.60/38.20	11.30/9.60	2.80/3.50	2.70/11.30
**Group 2** (0.5–1 year) N = 6	E-26/YC	10 months–1 year	-16.83	11.53	35.90	10.60	3.40	10.20
	E-34/YC	6 months–1 year	-22.80	11.00	37.20	12.00	3.10	8.30
	E-46/YC	6 months–1 year	-18.33	14.88	36.10	10.60	3.40	10.30
	CCC(3)/YC	1 year	-22.51	10.92	31.40	9.50	3.30	6.70
	C(293)/YC	6 months–1 year	-19.68	11.35	36.00	10.60	3.40	10.30
	C(295)/YC	6 months–1 year	-18.96	11.03	35.80	10.80	3.30	9.20
**Mean** **(SD)**			-19.85 (2.37)	11.79 (1.53)	35.4 (1.80)	10.68 (0.80)	3.30 (0.10)	9.20 (1.30)
**Min/Max**			-22.80/-16.80	10.92/14.88	31.40/37.20	9.50/12.00	3.10/3.40	6.70/10.30
**Group 3** (1–2 years) N = 8	E-27/YC	1.5–2 years	-16.48	10.90	35.90	10.60	3.40	10.50
	E-35/YC	1.5 years	-17.20	12.05	39.50	11.00	3.60	13.70
	E-42/YC	1–1.5 years	-18.77	14.17	38.50	11.70	3.30	11.90
	E-44/YC	1–1.5 years	-22.28	9.18	35.90	10.90	3.30	10.20
	E-45/YC	1.5–2 years	-18.29	12.99	36.30	10.10	3.60	11.10
	E-53/YC	1–1.5 years	-17.40	11.31	39.20	11.90	3.30	12.80
	E-54/OC	1.5–2 years	-15.80	12.00	35.80	11.50	3.10	10.00
	CCC(2)/YC	1.5–2 years	-20.80	13.36	34.90	11.60	3.00	9.10
**Mean** **(SD)**			-18.38 (2.20)	12.00 (1.57)	37.00 (1.70)	11.16 (0.62)	3.30 (0.20)	11.20 (1.40)
**Min/Max**			-22.28/-15.80	9.18/14.17	34.90/39.50	10.10/11.90	3.00/3.60	9.10/13.70
**Group 4** (2–6 years) N = 6	E-1/YC	3.5 years	-21.50	4.07	32.40	10.80	3.00	5.30
	E-28/YC	3–3.5 years	-16.47	12.72	37.80	11.10	3.40	13.80
	E-58/YC	3.5–4 years	-23.28	7.02	35.90	10.30	3.50	10.20
	E-64/YC	3–3.5 years	-25.61	7.02	36.20	10.60	3.40	7.30
	E-65/YC	2.5–3 years	-19.28	12.99	35.20	10.40	3.40	9.30
	C(4)/YC	5–5.5 years	-19.61	9.65	36.30	11.00	3.30	11.50
**Mean** **(SD)**			-20.96 (3.23)	8.91 (3.53)	35.60 (1.60)	10.70 (0.32)	3.30 (0.20)	9.60 (2.80)
**Min/Max**			-25.61/-16.47	4.07/12.99	32.40/37.80	10.30/11.10	3.00/3.50	5.30/13.80
**Adult females** **N = 18**	E-19b/OC	Young Adult	-13.03	8.72	31.90	10.60	3.00	6.10
	E-21/OC	Young Adult	-13.10	12.10	32.70	10.50	3.10	7.30
	E-23/OC	Young Adult	-13.88	8.99	35.60	12.30	2.90	5.20
	E-87/OC	Full Adult	-14.30	10.90	34.10	10.70	3.20	9.00
	E-88/OC	Young Adult	-15.70	10.10	31.90	10.60	3.00	7.40
	E-4/YC	Young Adult	-13.00	11.40	34.10	10.70	3.20	11.00
	E-5/YC	Full Adult	-14.80	11.40	37.50	10.40	3.60	12.20
	E-10/YC	Full Adult	-14.10	9.20	33.10	10.70	3.10	7.10
	E-24/YC	Full Adult	-10.50	11.50	32.60	10.50	3.10	10.90
	E-69/YC	Young Adult	-14.30	11.30	38.60	10.70	3.60	13.00
	E-70/YC	Young Adult	-16.30	11.40	37.20	10.60	3.50	12.20
	E-74/YC	Mature Adult	-12.60	10.90	30.80	10.60	2.90	6.30
	E-75/YC	Young Adult	-14.29	9.37	32.90	9.70	3.40	10.30
	E-81/YC	Full Adult	-14.00	11.20	34.10	10.70	3.20	8.20
	E-82/YC	Mature Adult	-17.27	12.83	38.70	12.10	3.20	11.40
	E-84/YC	Young Adult	-18.80	10.80	35.20	10.70	3.30	9.20
	E-94/YC	Mature Adult	-15.37	11.84	34.20	11.00	3.10	10.90
	E-95/YC	Mature Adult	-15.33	12.45	33.30	11.10	3.00	9.90
**Mean** **(SD)**			-14.48 (1.87)	10.91 (1.19)	34.40 (2.30)	10.79 (0.59)	3.20 (0.20)	9.30 (2.30)
**Min/Max**			-18.80/-10.50	8.72/12.83	30.80/38.70	9.70/12.30	2.90/3.60	5.20/13.00

Individuals were selected based on a minimum of nitrogen and carbon concentrations of 4.8% and 13%, respectively [36] and C/N atomic ratios within the range 2.9 to 3.6 (Table 1). This ensured minimal to no occurrence of bone collagen digenesis [37–38].

Juvenile ages were estimated using dental eruption [39] and long bone lengths [40–41]. The stages of development/senescence of the Canímar Abajo juveniles and adult females were determined using the [42] life history model. For the SIAR model analyses, juveniles were divided into four age groups: Group 1 (0 to 0.5 years), Group 2 (0.5 to 1 year), Group 3 (1 to 2 years) and Group 4 (2 to 6 years) in order to estimate dietary changes over time, and isotopic shifts that may be related to the introduction of weaning supplements. Sex of the adult individuals was determined using standard morphometric methods for pelvic, cranial, and post cranial remains [39, 43].

### Isotopic analyses

Collagen extraction was completed using the method described by DeNiro and Epstein [44]. According to the protocol previously mentioned, samples were demineralised in 1M HCl at 4°C for 1h while being stirred (every 5 minutes). After the samples were rinsed to neutral pH (de-ionised H_2_O), 0.2 M NaOH was added to remove contaminating humic acids and the samples were rinsed once more to neutral pH. Hydrochloric acid was added to the samples to obtain a pH of 2.5 and then covered to gelatinise at 70°C for 16 hours. Finally, the insoluble residues were removed by centrifuging the samples at 2500 r.p.m for 10 minutes and the remaining supernatant solution was evaporated at 100°C for 6 hours (until 3 ml remained). The solution was then freeze-dried for 24 hours.

Carbon and nitrogen elemental compositions and isotopic analyses of the bone collagen samples were accomplished by continuous flow isotope ratio mass spectrometry (CF-IRMS), performed at the University of Winnipeg Isotope Laboratory (UWIL). Collagen samples (0.2 to 0.6 mg) and internally calibrated standards were loaded into tin capsules and placed in an elemental analyser auto-sampler. Isotope results are expressed as “δ” values, which represent deviations in permil (‰) relative to the internationally recognized VPDB and Air standards for carbon and nitrogen, respectively. The carbon and nitrogen elemental compositions have analytical uncertainties of ± 0.5% each and the collagen carbon (δ^13^C_col_) and nitrogen (δ^15^N) isotopic compositions (values) were determined to have analytical uncertainties of ± 0.2 and 0.3 ‰, respectively (based upon a standard—sample ratio of 1:3; duplicate and triplicate sample repeats were run every 5^th^ and 10^th^ samples, respectively).

### Bayesian probability model considerations

Two models were used for estimating weaning ages: the WARN model (Weaning Age Reconstruction with Nitrogen isotope analysis [19]) and the SIAR model (Stable Isotope Analysis in R [20, 25–27]). Weaning period was estimated using the WARN model [19] which provides both start and end age estimates of weaning within the framework of approximate Bayesian computation (ABC). The WARN model is unique because it accounts for changes in the turnover rates of bone collagen, which is high during the first year of life and decreases until puberty. In this approach, the juvenile δ^15^N values are compared against the nitrogen isotopic enrichment between juveniles and females, and the δ^15^N values synthesized entirely from weaning foods. This allows an estimation of their age at the start and the end of the weaning process (within the ABC framework).

Possible food sources and probabilistic changes in source importance over time, were assessed by using the SIAR model [20, 25–27]. To estimate importance of the breast milk in juvenile diets, carbon and nitrogen isotopic compositions of Canímar Abajo female breast milk was inferred, and included as a food source in the model. To infer the female breast milk isotopic compositions, we calculated the carbon and nitrogen variations between collagen and milk by combining the relationships between mother’s milk and hair [45–46] and hair to collagen [47, 48] (Table 2).

**Table 2:** δ^13^C and δ^15^N isotopic offsets between maternal bone collagen and maternal milk; and maternal milk–infant bone collagen.

Isotopic offset	δ^15^N_col offset_ (‰)	δ^13^C_col offset_ (‰)	Reference
Maternal milk- maternal bone collagen^a^	3.6–3.44	4.58–5.55	Δ keratin-milk [45, 46]; Δ keratin–collagen [47, 48]
Milk diet–infant bone collagen^b^ For exclusive breastfeeding infants	4.50–4.34	4.98–5.93	Δ keratin-milk [46]; Δ keratin–collagen [47, 48]

*We define: Δ _a-b_ = δ^15^N_b_—δ^15^N_a_; Δ _a-b_ = δ^13^C_b_—δ^13^C_a._ Therefore, Δ milk-collagen = δ^15^N_col_—δ^15^N_milk_ and Δ milk-collagen = δ^13^C_col_—δ^13^C_milk._

^a^ Calculated from the relationship keratin-milk: -2.58‰ and -4.12 for N and C, respectively [45, 46] and keratin-collagen: from 0.86 to 1.02 and from 0.46 to 1.41 for N and C, in that order [47, 48].

^b^ Calculated from the relationship mother milk-newborn’s hair: 3.48 and 4.52 for N and C, respectively [46] and hair-collagen [47, 48].

Based on the food source evidence, we made assumptions regarding the types of breast milk supplements available and subsequently used for the Canímar Abajo juveniles. Given the lack of animal milk in the pre-contact Caribbean, we assume that babies would be initially weaned on soft vegetable foods that were available to the Canímar Abajo populations: such as legumes, root cultigens, possibly maize [35] and tropical fruits. The use of maize is considered unlikely as the carbon and nitrogen isotopic composition of Canímar Abajo juveniles suggested that only C_3_ plant based terrestrial resources were used as weaning foods [5] (also see Table 1). Therefore, the average carbon and nitrogen isotopic compositions (and respective standard deviations) of plant supplements were included in the SIAR mixing model [20]. Isotopic values of the sources were taken from Pestle [4] and cited literature sources (S1 Table).

We also included the concentration dependencies of breast milk and vegetable sources, along with the ratio diet-collagen for each specific source, in the SIAR mixing model (Table 3). Breast milk carbon and nitrogen concentration dependencies were taken from Romek and colleagues [46] and differences between milk and collagen were estimated from the differences between mother’s milk and infant hair [46] and hair to collagen [47,48] (Table 2). The concentration dependencies of C_3_ plant resources included in the model were taken from Pestle [4] and the cited literature sources (S1 Table), while the diet-collagen differences were obtained from Chinique de Armas and colleagues [35] (Table 3; S2 File). These values, in association with Canímar Abajo juvenile δ^13^C_col_ and δ^15^N data (Table 1), resulted in a percent mode, and low and high 95% credibility intervals, for each of the supplement sources of protein considered.

**Table 3:** Isotopic compositions, concentration dependencies and diet-collagen fractionations ratios of the source diet components used for the SIAR model calculations.

Source	Source Isotopic Compositions (‰)^a^				Concentration Dependences (%)^b^				Diet-Collagen Fractionations (‰)^c^			
	δ^15^N_source_	Sd	δ^13^C_source_	Sd	Nitrogen	Sd	Carbon	Sd	∆δ^15^N_diet-col_	Sd	∆δ^13^C_diet-col_	Sd
**Breast Milk**	7.39	1.19	-19.55	1.87	1.61	0.27	49.13	3.63	4.42	0.11	5.46	0.67
**Root Cultigens**	3.84	1.56	-22.45	1.21	1.09	0.09	41.36	1.34	2.00	0.20	4.40	0.90
**Legumes**	2.23	1.23	-22.86	0.83	3.82	0.33	41.78	0.41	2.00	0.20	4.40	0.90
**Tropical Fruits**	3.54	2.14	-23.06	1.92	1.37	0.61	43.46	4.50	2.00	0.20	4.40	0.90

^a^ S1 Table shows the data base used for infering the isotopic composition of non-milk sources

^b^ [C] and [N] porcentages were taken from Romek et al. [46] (milk) and Pestle [4] and cited literature (non-milk sources) (S1 Table).

^C^ Diet -collagen fractionations considerations for non-milk sources are in S2 File. Diet-Collagen fractionation ratio (milk) is for exclusively breastfeeding infants. Values are mean values and SD of the ranges presented in Table 2. ‰ Air for nitrogen; VPDB for carbon.

According to the Shapiro’s test, all sets of isotope data are normally distributed (C: W = 0.97, *p* = 0.53; N: W = 0.99, *p =* 0.90). One-way ANOVA with a Tukey-Kramer post-hoc test was used in assessing carbon and nitrogen isotopic means among the different juvenile age groups and between females and the different age groups. A statistical significance was set at α = 0.05 level.

## Results

**Fig 2:**
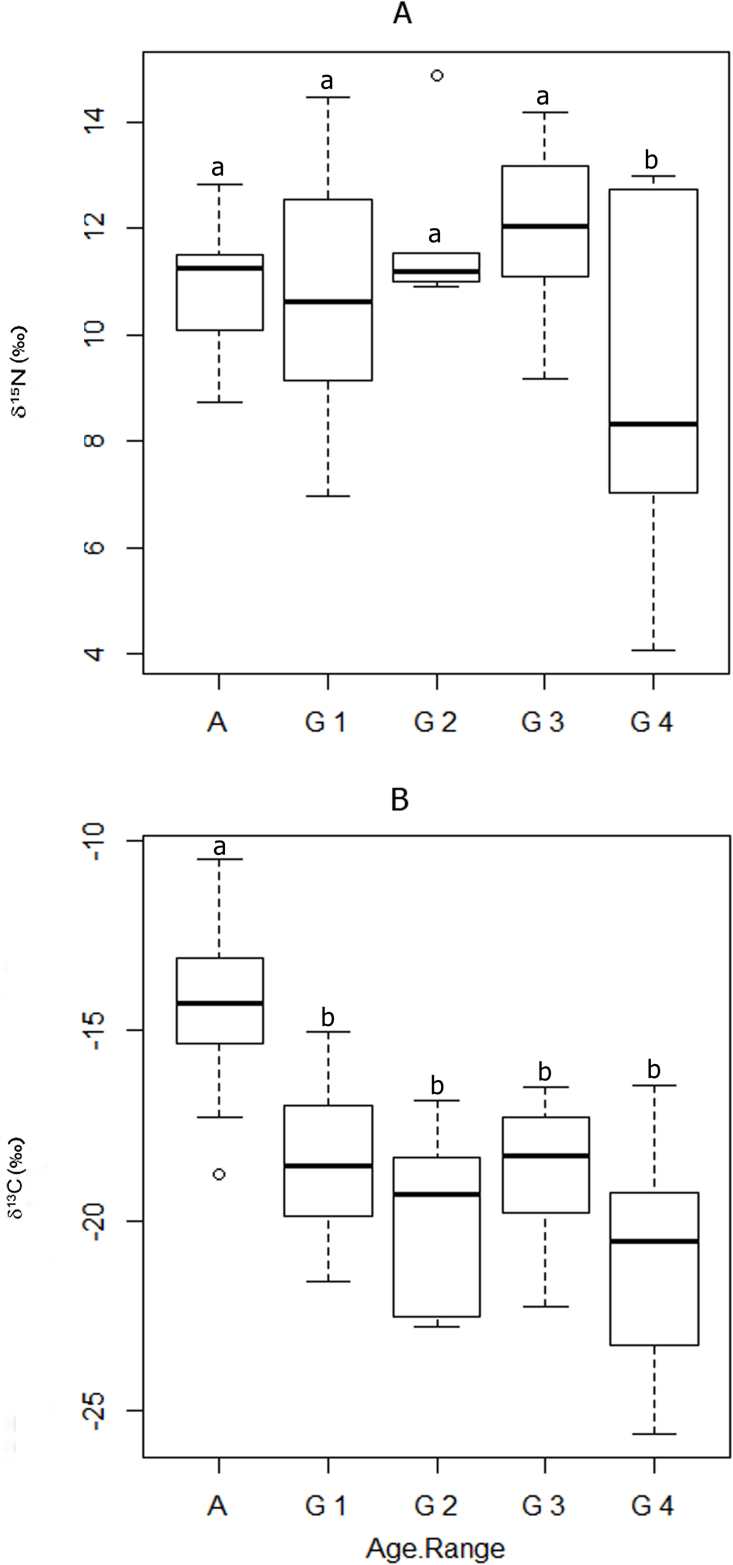
δ^15^N and δ^13^C_col_ isotopic values among age ranges. Different letters show statistically significant differences. A: Adult, G 1: Group 1, G 2: Group 2, G 3: Group 3, G 4: Group 4.

Nitrogen and carbon isotope values for Canímar Abajo females and juveniles are provided in Table 1. The only statistically significant nitrogen isotope difference in means was observed between Group 3 (1 to 2 years) and Group 4 (2 to 6 years) (p = 0.043; Table 4; Fig 2A). Table 4 also indicates that means of Group 2 (0.5 to 1 years) and Group 3 (1 to 2 years) are similar, as is the case for the means of females and juveniles from the first two groups (infants between 0 and 1 year old) (Fig 2A).

**Table 4:** Results of the Tukey Kramer multiple comparisons of isotope means among juvenile age groups and female individuals. Group 1 (0 to 0.5), Group 2 (0.5 to 1), Group 3 (1 to 2) and Group 4 (2 to 6). Diff.: difference. ANOVA: N: F = 2.45, df = 4; C: F = 14.5, df = 4; p≤0.05.

δ^15^N	Diff	Lower	Upper	p
**Group 1—Group 2**	0.965	- 1.88	3.813	0.870
**Group 1—Group 3**	0.194	- 1.413	3.801	0.691
**Group 1—Group 4**	-1.902	- 4.750	0.946	0.333
**Group 2—Group 3**	0.229	-2.801	3.259	0.999
**Group 2—Group 4**	-2.867	-6.106	0.373	0.105
**Group 3—Group 4**	-3.096	-6.126	-0.066	**0.043**
**Female—Group 1**	0.093	-2.054	2.240	0.999
**Female—Group 2**	-0.872	-3.517	1.773	0.880
**Female—Group 3**	-1.101	-3.485	1.283	0.684
**Female—Group 4**	1.994	-0.650	4.639	0.220
**δ**^**13**^**C**				
**Group 1—Group 2**	-0.929	-4.148	2.290	0.923
**Group 1—Group 3**	0.355	-2.593	3.302	0.997
**Group 1—Group 4**	-2.212	-5.431	1.007	0.305
**Group 2—Group 3**	1.283	-2.142	4.709	0.823
**Group 2—Group 4**	-1.283	-4.945	2.379	0.855
**Group 3—Group 4**	-2.567	-5.992	0.859	0.226
**Female—Group 1**	4.271	1.844	6.699	**0.000**
**Female—Group 2**	5.200	2.210	8.190	**0.000**
**Female—Group 3**	3.916	1.221	6.612	**0.001**
**Female—Group 4**	6.483	3.493	9.473	**0.000**

Statistically significant carbon isotope differences were detected between Canímar Abajo females and each of the juvenile groups (Table 4; Fig 2B). Among juveniles, major similarities in carbon mean values exist among groups 1 (0–0.5 y), 2 (0.5–1y) and 3 (1–2 y). Although not statistically significant, there is a low probability that the mean values of carbon for groups 1 and 3 are similar to carbon values of group 4 (2–6 y) (Table 4; Fig 2).

### WARN model: Ages at the start and end of weaning

**Fig 3:**
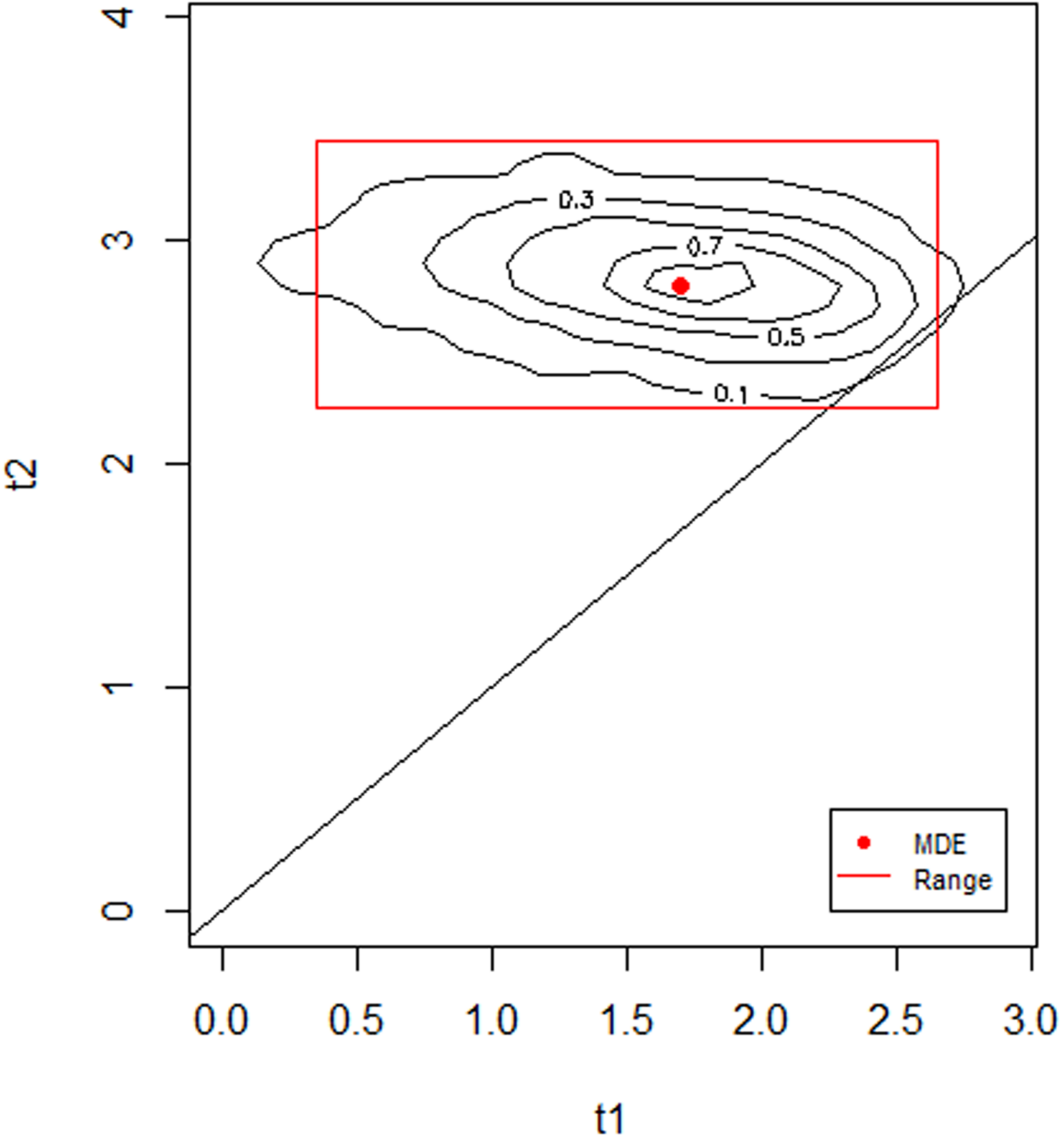
WARN model probability and 95% credibility interval of beginning (t_1_) and ending (t_2_) ages of weaning for the Canímar Abajo juveniles.

WARN model estimations (95% credibility interval) suggested that the start of weaning (t1) occurred between 0.4 and 2.6 years while the end of weaning (t2) took place between 2.3 and 3.4 years of age (Fig 3). There is a higher probability for the start of weaning to have occurred close to one year and 9 months of age (maximum density estimate MDE—1.7 years), while weaning was likely completed by approximately 3 years (MDE– 2.8 years) (Fig 3). Enrichment of δ^15^N in juvenile relative to female collagen is in the range between 0.3 to 1.7 ‰ (MDE: 1.0 ‰) and collagen derived entirely from weaning foods range from 6.0 to 8.8 ‰ (MDE: 7.4 ‰).

**Fig 4:**
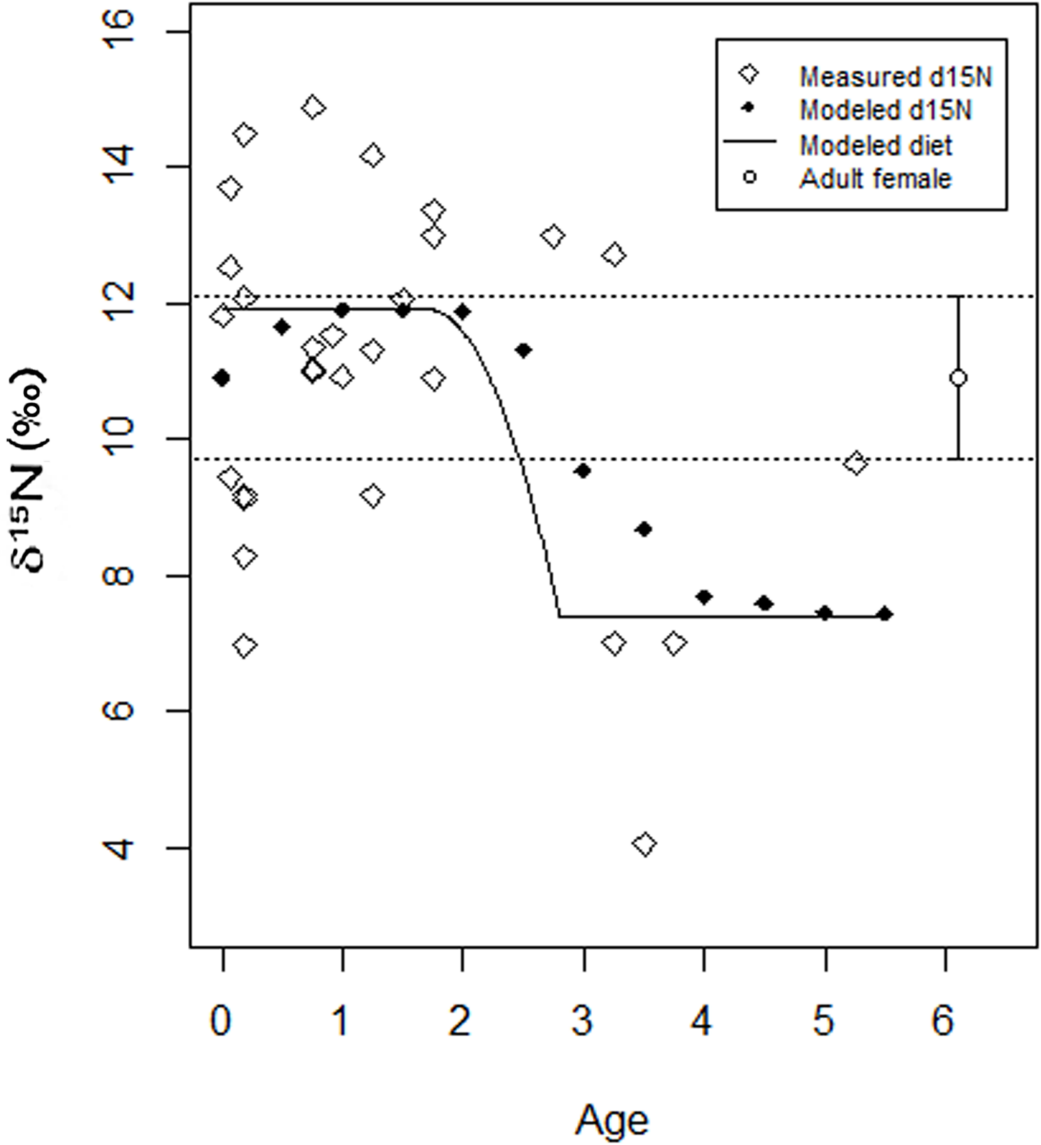
Transverse comparison of δ^15^N (‰ Air) values by juveniles age calculated from reconstructed maximum density estimators (MDEs) [19]. Mean and SD range for adult females and all juveniles are indicated with open circles and crosses, respectively.

Modeled differences in the δ^15^N values by juvenile ages, calculated from reconstructed MDEs [19], suggest that nitrogen isotope values were more enriched for juveniles that were around 1 year of age, than for ones older than 2 years (Fig 4). Measured nitrogen isotope values are particularly depleted for some juveniles who are between 3 and 4 years of age. The only juvenile in the sample greater than 5 years of age, had a measured nitrogen isotope value close to the mean female nitrogen isotope value (Fig 4).

### SIAR model: Important of dietary sources by ages

The SIAR model estimations (95% credibility interval) of the relative importance of different sources by age groups are summarised in Table 5.

**Table 5:** SIAR model percent mode estimates and accompanying high (HCI) and low (LCI) 95% credibility intervals (CI) for potential diet protein sources for the Canímar Abajo 0–1, 1–2 and 2–5 year old juveniles.

Food Sources	Range 1 (0–0.5 y)			Range 2 (0.5–1 y)			Range 3 (1–2 y)			Range 4 (2–6 y)		
	Mode	95% CI		Mode	95% CI		Mode	95% CI		Mode	95% Cl	
		LCI	HCI		LCI	HCI		LCI	HCI		LCI	HCI
**Breast Milk**	57	27	83	37	2	88	77	23	92	34	4	60
**Root cultigens**	4	0	35	14	0	43	3	0	34	31	0	53
**Legumes**	1	0	20	1	0	32	1	0	28	3	0	37
**Tropical Fruits**	20	0	46	3	0	44	4	0	43	27	0	44

#### Group 1 (0 to 0.5 years old)

**Fig 5:**
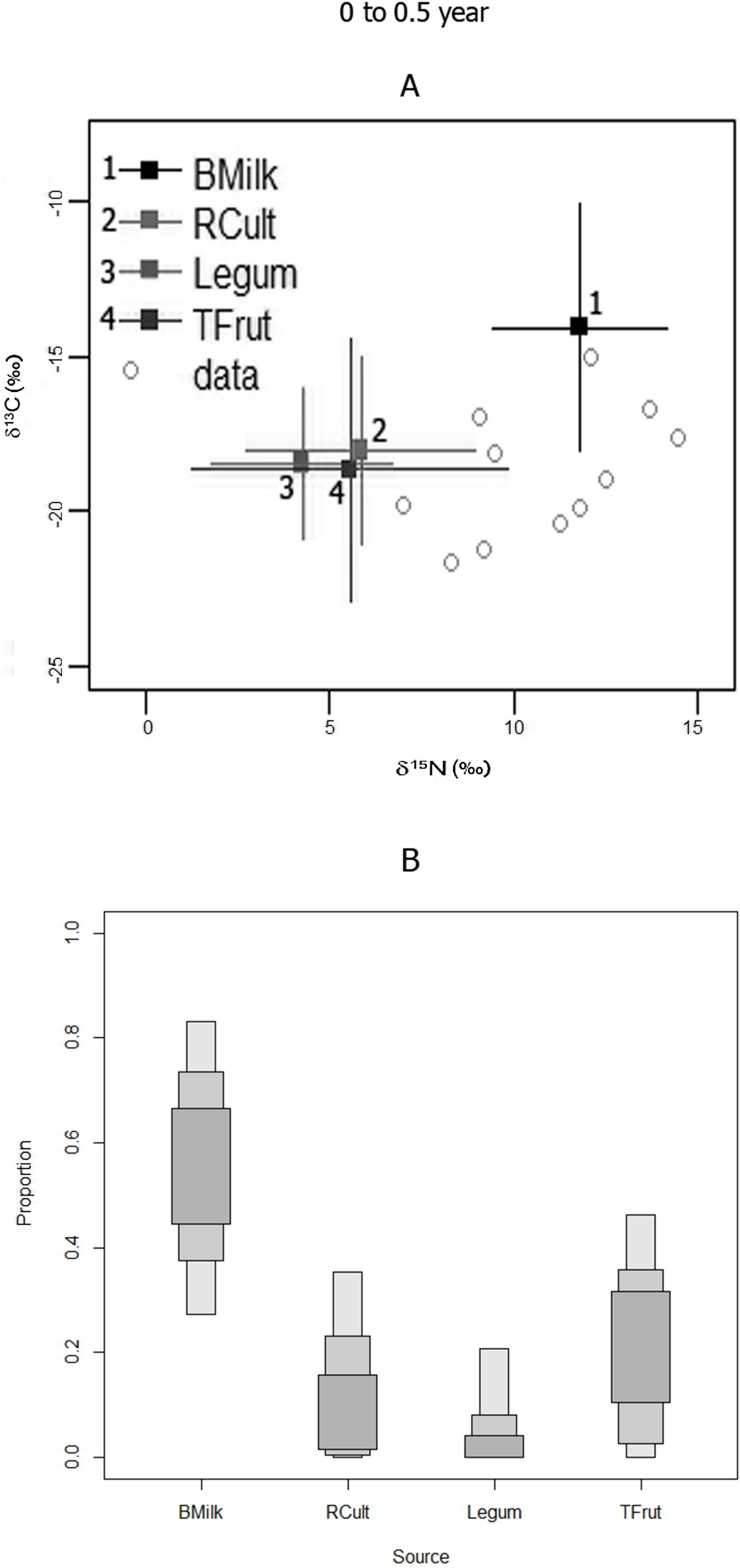
**GROUP 1 (0–0.5 years):** A) SIAR model isotopic distributions of juveniles bone nitrogen and carbon with respect to possible food sources; B) SIAR model percent mode estimates and accompanying high and low 95% credibility intervals (error bars) for breast milk and vegetable sources for the Canímar Abajo juveniles.

The model indicates that breast milk was the source that contributed the most to Group 1 diets (Fig 5). However, there is variability in nitrogen and carbon isotopic values within the group (Fig 5A). There is some possibility that other food sources, especially tropical fruits, may have supplemented 0–0.5 year old juvenile diets (see mode and HCI in Table 5). There is a higher probability that supplements were not used for the majority of the juveniles, as the modes are comparatively low, for all considered supplements (note the zero low credibility interval for the supplements) (Table 5, Fig 5).

#### Groups 2 (0.5 to 1 year old) and Group 3 (1 to 2 years old)

**Fig 6:**
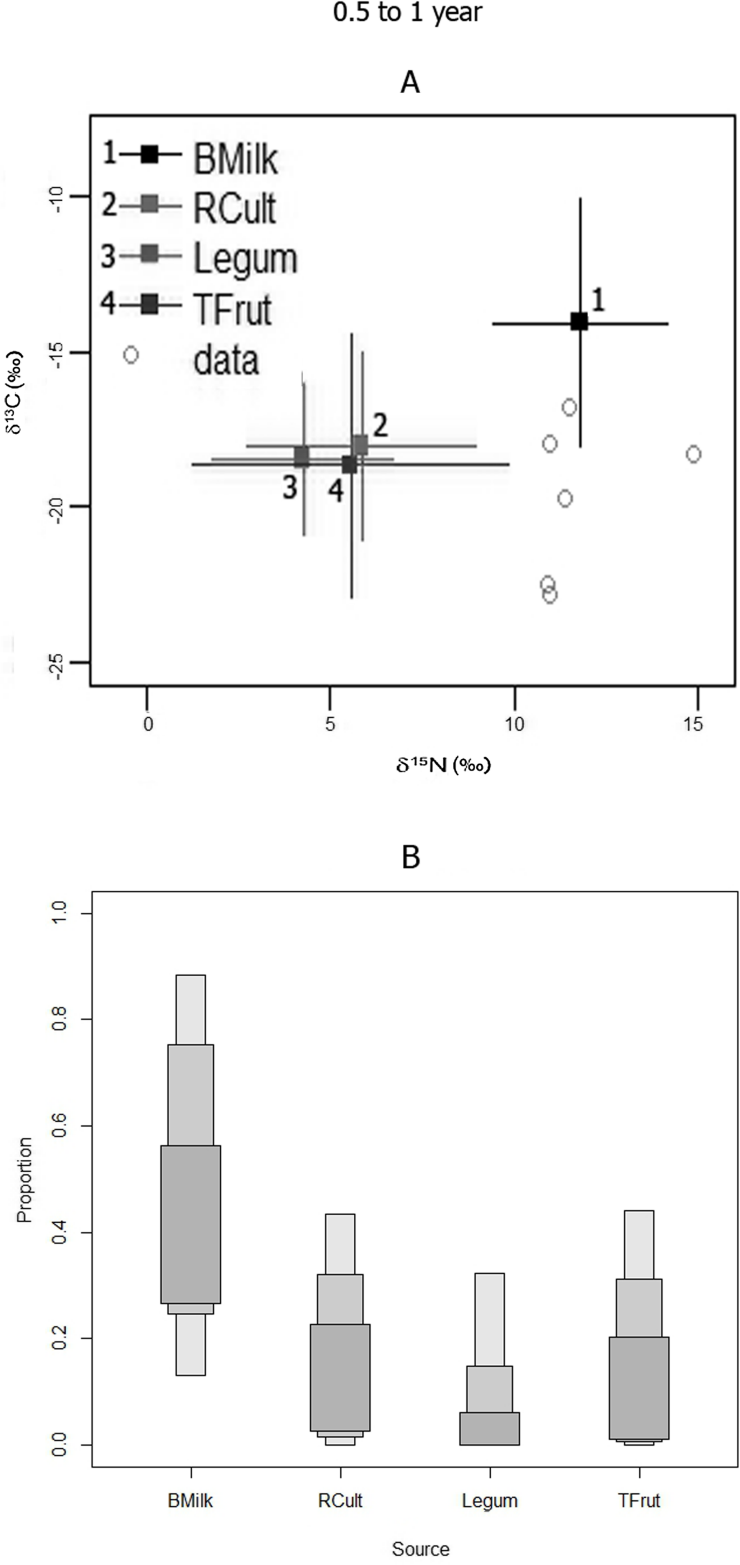
**GROUP 2 (0.5–1 year):** A) SIAR model isotopic distributions of juveniles bone nitrogen and carbon with respect to possible food sources; B) SIAR model percent mode estimates and accompanying high and low 95% credibility intervals (error bars) for breast milk and potential supplement protein sources for the Canímar Abajo juveniles.

**Fig 7:**
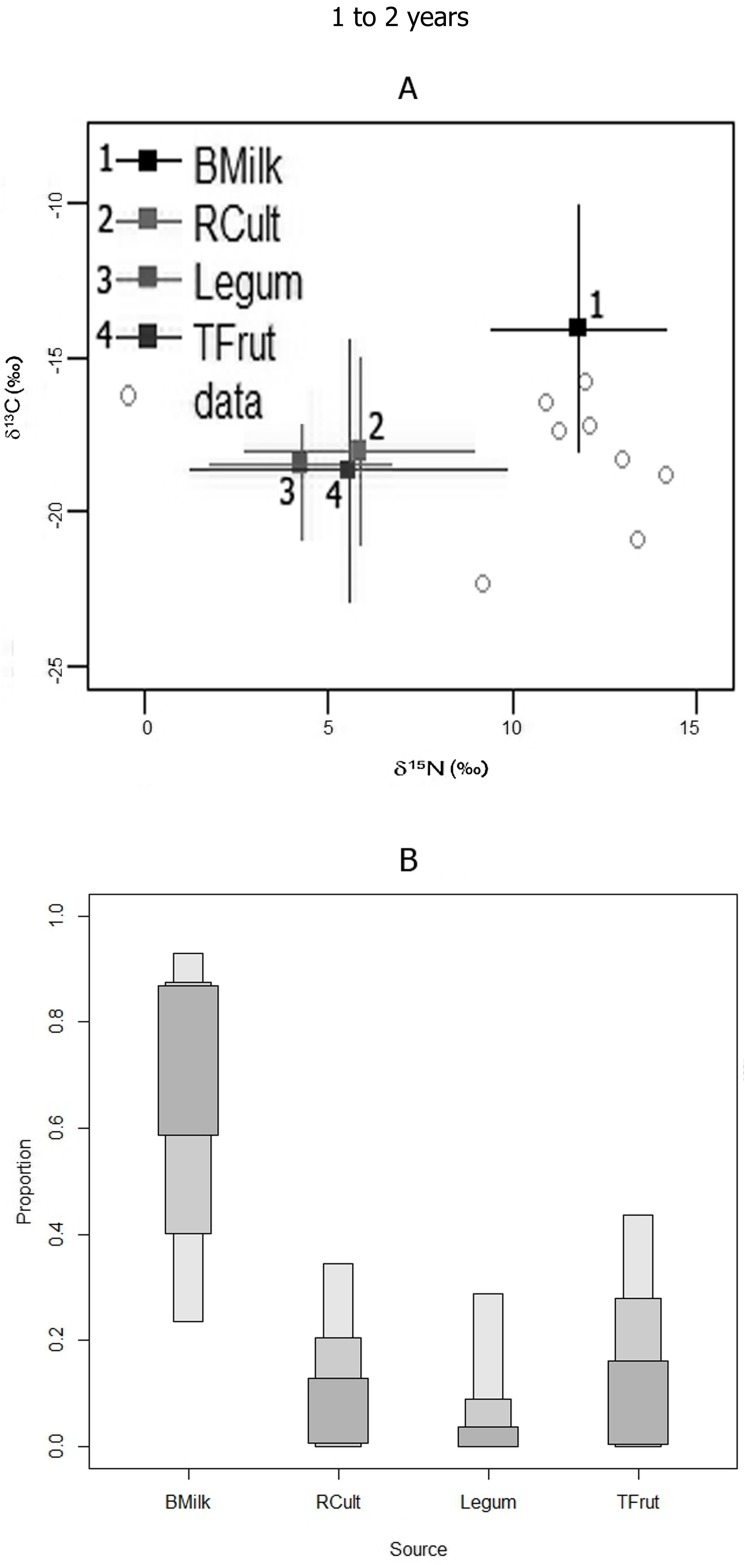
**GROUP 3 (1–2 years):** A) SIAR model isotopic distributions of juveniles bone nitrogen and carbon with respect to possible food sources; B) SIAR model percent mode estimates and accompanying high and low 95% credibility intervals (error bars) for breast milk and potential supplement protein sources for the Canímar Abajo juveniles.

According to the SIAR model analyses, breast milk contribution to diet was higher, relative to other food sources, for both Group 2 (0–0.5 y) and Group 3 (1–2 y) juveniles (Table 5, Figs 6 and 7). The breast milk component is inferred to be greater for the Group 3 relative to the Group 2 (Table 5). Also, there is a slightly higher probability for Group 2 to have consumed substantial amounts of other supplements such as root cultigens (Table 5, Figs 6 and 7). However, it is also possible that these supplements were not present, or had low importance, in most of Group 1 and 2 juveniles’ diets because their LCI is zero, and the modes are lower than breast milk, particularly for Group 3 juveniles (Table 5).

#### Group 4 (2 to 6 years old)

**Fig 8:**
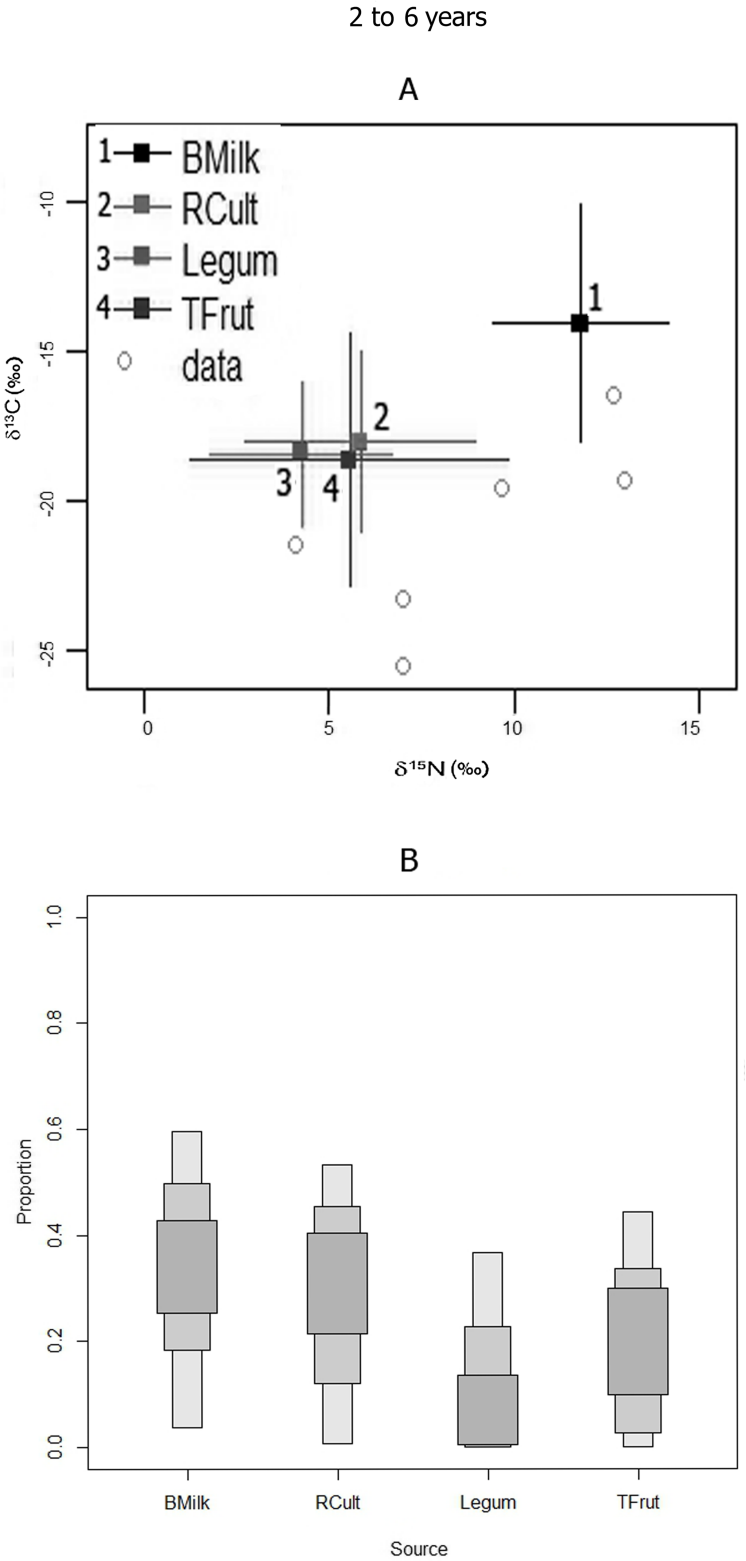
**GROUP 4 (2–6 years):** A) SIAR model isotopic distributions of juveniles bone nitrogen and carbon with respect to possible food sources; B) SIAR model percent mode estimates and accompanying high and low 95% credibility intervals (error bars) for breast milk and potential supplement protein sources for the Canímar Abajo juveniles.

The SIAR probability model indicates that the greatest evidence for reduction of breast milk importance, relative to other supplements, occurred for Group 4 (2 to 6 y) juveniles. The mode value for breast milk intake is lower, with a LCI of only 4% (Table 5). Supplements such as root cultigens, tropical fruits and legumes constitute much higher possible protein sources, at least for part of the population (note population variation in Fig 8A), with significantly higher credibility intervals (Table 5, Fig 8).

## Discussion

Both the WARN and SIAR models suggested that breast milk was the main source of dietary protein for most Canímar Abajo juveniles during the first two years of life, with the possibility of supplementary sources, such as tropical fruits and root cultigens, being part of some juvenile diets by six months of age or even earlier. A child’s first months/years of life require high nutrition for rapid growth and development, and there are limitations on how nutrients are ingested due to their underdeveloped gastrointestinal tracts [49]. Compared to other types of food, breast milk is easily digestible. It also contains most of the essential nutrients a child needs for healthy early development, such as fat, carbohydrates, minerals, proteins, vitamins, water and immunoglobulin [50].

Information on current populations suggests that by six months, breast milk does not fully satisfy a child’s nutritional needs [51–52]. At this stage, in general, a child’s digestive tract is usually mature enough to digest more complex foods [52]. However, while not entirely sufficient, breast milk still remains an important source of energy, nutrients, and anti-infective factors lowering infant mortality and morbidity [53, 54]. Nitrogen isotopic compositions vary widely in the first year of life, suggesting that some individuals were weaned before others. This pattern of variation during the first year has been observed previously in other non-industrial societies [8, 18].

Some infants younger than six months have very low nitrogen values. Two different possibilities can explain this scenario: 1) they were already weaned which could be related to their early death or, 2) the breast milk isotopic signal has not been completely fixed in their bones, as suggested by the apparent similarity between female nitrogen values and juveniles from groups 1 and 2. If they were not being breastfed at the time of death, tropical fruits may have been used as alternative foods which is possible according to the SIAR model calculations and the depleted carbon isotopic composition of some juveniles.

SIAR analyses indicate a higher dependence on breast milk for Group 3 (1 to 2 years) than for Group 2 (0.5 to 1 year) juveniles. This suggests that, if supplementary foods were introduced to some individuals at six months of age or earlier, the juveniles who were exclusively breastfed may have had a greater survival rate than partially or completely weaned juveniles and, as such, are better represented in Group 3 (1 to 2 years). This is consistent with previous results suggesting that mortality and morbidity is lower for exclusively breastfeed infants. In fact, under more challenging living conditions (poor hygiene, crowding, lack of adequate care, heavy pathogen load, etc.), the importance of exclusive breastfeeding for infant survival seems to be higher [6].

This study relies on cross-sectional data, and therefore, it is not possible to assess changes in breast milk consumption as these infants matured. Furthermore, statistics reconstructed from archaeological skeletal series do not provide actual health status and life history information of past populations, because there are individual levels of heterogeneity in the risk of death and disease, and because the skeletal series represent only dead juveniles who do not survive past the age at which they were examined [55–56]. Future longitudinal isotopic studies using analysis of tooth dentine and enamel of Canímar Abajo adults—individuals who survived the weaning process–will provide more information and will allow us to deal with this osteological paradox.

The WARN model results suggest that the weaning process ended just prior to 3 years of age. These results indicate that the population of Canímar Abajo weaned their children earlier than the agricultural population of Paso del Indio in Puerto Rico, where the WARN model suggested that weaning commenced around 3 years of age (MDE: 2.7 y) and was completed by six years of age (MDE: 5.9 y) [57]. This supports recent results suggesting weaning did not necessarily occur earlier (and was of shorter durations) among farmers compared to foragers [19, 58]. Alternatively, the Canímar Abajo population may have been more dependent on cultivated food than is currently recognized [35].

The introduction of supplementary food is taken to be the time when nitrogen isotope compositions start to deplete [13–14, 18, 56]. As discussed previously, both the SIAR and the WARN model analyses concur upon a higher probability age of about 2 years, for the introduction of supplementary food for Canímar Abajo juveniles. If the start of weaning was between 1.5 and 2 years of age, then the introduction of supplementary food in the Canímar Abajo population would be later than those reported for ethnographic populations [19] and, also, a year and a half later than current recommendations for proper nutritional requirements (supplementation at 6 months of age). However, it is important to remember that these requirements are not necessarily met by protein.

The δ^15^N values reflect the ratios of dietary protein, because nitrogen mainly originates from amino acids [59–60]. Ethnographic studies have indicated that liquids such as fruits, teas, root cultigens and cereal gruel, with lower protein contents, are frequently used in the weaning process of modern and traditional human populations [7, 58], with high protein foods often being avoided as supplementary foods [61]. Therefore, the actual age when solid or liquid foods were introduced as supplements could be lower, as suggested by the wide probability range (95% credibility interval) for the start of weaning indicated in Fig 3 and the SIAR model. As observed in other archaeological populations [19], juvenile δ^15^N values did not record this type of change.

It is not possible to determine beyond doubt the age at which Canímar Abajo populations actually introduced the first non-milk foods to their juveniles. Juvenile carbon isotopic compositions are depleted in comparison to female adults in all ages ranges (Fig 2), supporting the idea of supplementation since early ages. It is likely that between 1.5 and 2 years of age, a continuous introduction of supplementary food occurred, and sources such as root cultigens, legumes and tropical fruits (or food with similar nitrogen isotopic values as most terrestrial resources), gained importance in the juvenile diets relative to breast milk. A zero LCI value in the SIAR model, for non-milk food after 2 years of age, is likely due to the fact that juvenile bone collagen turnover rates decrease with age; and consequently, bones take longer to reflect isotopic changes in dietary input [19]. The statistically significant difference for nitrogen isotopes (rapid depletion of δ^15^N values) between Group 3 (1 to 2 years) and Group 4 (2 to 6 years) juveniles, suggests a rapid transition between food sources, which is typically observed when the weaning period is brief [19].

The early supplementation based on low protein resources (liquid fruits or root cultigens) is supported by the low nitrogen isotope enrichment observed between females and juveniles from the Group 1 and 2 age ranges. According to Tsutaya and Yoneda [56] this happens when there is a short period of exclusive breastfeeding where nitrogen isotopes in juvenile tissue protein do not fully equilibrate with breast milk, and therefore, still reflect depleted nitrogen isotopic signals from other sources. Furthermore, infant mortality for Canímar Abajo juveniles between 1 and 2 years of age is higher than for other juvenile age groups [62], a phenomenon that could be associated with the weaning process. Introduction of liquids and supplementary foods to juvenile diets can increase the risk of mortality [12, 63–64]. Therefore, by accounting for possible lag times in bone collagen isotopic signal imprinting (discussed above), it seems possible that some juveniles between 1 and 2 years of age could have been exposed to supplementary foods that contributed to their deaths.

By the age of three, δ^15^N and δ^13^C values of most juveniles are very depleted (more depleted than average female isotopic values–Figs 2 and 4), suggesting that transitional food sources with depleted nitrogen and carbon isotope values such as root cultigens, legumes and possibly marunguey (*Zamia* spp.) were predominant in their diet. Marunguey has very low nitrogen and carbon values and was identified in the dental calculus of the adults [35]. As indicated by the WARN model, the weaning process was likely completed by that time. In general, lower trophic level foods, such as root cultigens, cereals and legumes are preferred as weaning foods over higher trophic levels foods, such as terrestrial and marine/riverine animals [19, 58, 65]. Previous paleodietary studies identified wild and cultivated root species and legumes present in the Canímar Abajo populations (*Ipomoea batatas*, *Xantosoma* sp., *Phaseolus vulgaris*, *Zamia* spp. among others) [35, 66], indicating that these plants could have been used as supplemental and transitional weaning foods.

The only juvenile older than five years of age included in this study had a δ^15^N value close to some adult female values (Fig 4), suggesting that the child may have started to incorporate protein sources with higher nitrogen values. Canímar Abajo adult diets relied mainly on marine/riverine resources but included terrestrial animals and C_3_/C_4_ plants [5, 35]. By the age of five, children may have started to rely more in the type of proteins that formed part of the adult diet. Although it is not possible to infer that all Canímar Abajo children over 5 years of age had a nitrogen diet isotopically similar to the adult females, this is consistent with the weaning pattern described for the agricultural population of Paso del Indio in Puerto Rico, where older juveniles, once weaned, were fed with diets isotopically similar to those of the adults [4, 57]. However, in the case of Canímar, carbon isotopic values are still different between juveniles and adults (Fig 2), suggesting that juveniles were not fed with all foods used by the adult population.

Carbon mean isotopic values of juveniles from 0 to 2 years of age are similar, suggesting that they took advantage of sources with similar carbon isotopic compositions. Among them, the group where milk seemed to be less important related to other sources (group 2) showed a greater similarity with group 4 carbon isotope averages. This supports early supplementation and suggests that carbon isotope variations could be associated with the nature of weaning foods used, rather than with differences in the isotopic composition of female breast milk. It is important to take into account that carbon isotope values are variable for infants within the same age ranges. This variation indicates that sources with different carbon isotopic values (likely C_3_ plants and/or C_3_ plant-based animals) were being used as supplementary and transitional foods.

Juvenile mean carbon isotopic concentrations are statistically different from female adult values, which suggest that more enriched marine resources or C_4_ plants were likely not part of the juvenile diet. While female individuals had a mixed diet where resources enriched in δ^13^C (marine resources and C_4_ plants), were present [5, 35], juveniles seem to have relied mainly on terrestrial resources (depleted in δ^13^C). The fact that juveniles and adults do not differ significantly in nitrogen isotopic values could indicate that carbon isotopic differences are not only influenced by proteins but also by other macronutrients such as carbohydrates [60]. If this is the case, the main difference between adult and juvenile diets could be a differential consumption of plants, where C_4_ plants were essentially absent in the juvenile diets. Further studies involving a bivariate regression model with both collagen and apatite isotope data could be important for the understanding of the observed differences [60].

Studies of juvenile nutrition are rare for the Caribbean and it is impossible to ascertain whether the lack of maize in juvenile diets, as suggested here, was a wide spread phenomenon. While maize seems to have been consumed by juveniles in the circum-Caribbean area (*e*.g., juveniles from Mesoamerica) [8, 67], it seems to have been absent from the diet of juveniles from other Antillean sites [68]. Our results, while not conclusive, are consistent with a possible restriction of maize consumption among juveniles in (at least some) Caribbean aboriginal populations. More research on juveniles is required before we can explain the absence of maize in both Canímar Abajo juvenile diets and among juveniles of other Antillean populations where the plant was likely part of the adult diet [68].

Variations in SIAR model predictions are possible due to different reasons. For instance, the model uses the isotopic offset ‘diet-collagen’ (e.g., milk-collagen; other sources–collagen) to calculate probabilities of the relative importance of different sources in diet. Some studies have detected diet-specific variations in the diet-collagen fractionation factors, suggesting that the exact dietary offsets (Δδ^13^C_diet-col_ and Δδ^15^N_diet-col_) are variable and influenced by the isotopic composition of the nutrients consumed (for a further discussion of the topic see Froehle and colleagues [60]; [18]). Additionally, the value used in this paper to account for the difference between maternal milk and infant collagen could be more variable than expected. However, it is important to highlight that this model provides the opportunity of including non–fixed values for the considered ratios, which constitute an advantage relative to traditional approaches. Variations arising from using SIAR for breastfeeding and weaning practice reconstructions can also be related to the fact that the model does not account for bone turnover rates and that proportions can vary depending on the selected dietary sources. This means that the results obtained from the model should be considered as possibilities and not as a final evidence of the consumed sources or the timing of weaning. Nevertheless, by combining SIAR with the WARN model, which does not contain the uncertainties previously discussed, it is possible to confidently evaluate SIAR predictions. This approach allows the integrated results from two models, within a probabilistic framework, to obtain more robust information about the timing of weaning and the possible food sources used for an ancient population to wean their children.

## Conclusions

The combination of the results of two Bayesian probability models (WARN and SIAR) enabled the estimation of age at the start and the end of the weaning process in an ancient Caribbean population, while implementing more realistic isotope modeling parameters, such as non-static bone collagen to food isotope discrimination factors, protein contents for different sources and bone collagen turnover rates. The WARN model was useful in providing an estimate for the start and the end of weaning. Furthermore, it complemented the SIAR model weaning age results which used inferred breast milk isotopic compositions and also provided probabilistic changes in resource importance over time.

Results presented here suggest that milk was the main source of dietary protein for Canímar Abajo juveniles during their first two years of life. Some supplements, with low nitrogen isotopic values and protein contents, such as tropical fruits and root cultigens, were likely used to feed juveniles during this period; but the importance of these foods was probably low compared to breast milk. Around 2 years of age, supplements gained importance in Canímar Abajo juvenile diets. By then, C_3_ plants, including legumes, were probably continuously consumed as weaning foods. There appears to be a strong relationship between breastfeeding and the survival chances for juveniles, as suggested by a higher mortality for those infants whose diets were supplemented earlier in life.

At the age of three, the weaning process for the Canímar Abajo juveniles was likely completed. Subsequently, the juveniles were provided with a transitional diet, isotopically different from adult diets, consisting mainly of foods with more depleted carbon and nitrogen isotopic values such as root cultigens, legumes, and possibly marunguey. Maize and other resources used by the adults, such as marine/riverine animals with more enriched carbon and nitrogen isotopic values, appear not to have had contributed substantially to juvenile diets during their first years of life.

The combined use of the SIAR and WARN models helped to facilitate greater resolution in reconstructing the weaning process of the Canímar Abajo archaeological populations and, in doing so, may have established a new procedure for future isotope based weaning reconstructions.

## Supporting information

S1 Table Isotopic ratios of Caribbean terrestrial sources used for the SIAR model. (PDF)

S1 File Fig 1 use policy. (PDF)

S2 File Procedure used to account for the variation between diet and bone collagen. (PDF)
