# Supplementary material for: Isotopic reconstruction of the weaning process in the archaeological population of Canímar Abajo, Cuba: A Bayesian probability mixing model approach
Source: PLoS One. 2017 May 1;12(5):e0176065. doi: 10.1371/journal.pone.0176065 (PMC5411105; doi:10.1371/journal.pone.0176065)
Supplement: S1 Table — (PDF) [file pone.0176065.s001.pdf]

S 1 Table. Isotopic ratios of Caribbean terrestrial sources used for the SIAR model.

Source	δ^{13}C	δ ^{15}N	wt %C	wt %N	Sample type	Sample antiquity	δ $^{13}C_{adj}$ [a]	δ $^{15}N_{adj}$	Reference
**Root cultigens** (N=24)									
Dioscorea sp. (ñame)	-25.10	7.60			root	Modern	-21.10	7.60	Keegan and DeNiro (1988)
Dioscorea sp. (ñ. rojo)	-26.30	3.70			root	Modern	-22.30	3.70	Keegan and DeNiro (1988)
Dioscorea sp. (ñ. blanco)	-27.00	5.20			root	Modern	-23.00	5.20	Keegan and DeNiro (1988)
Dioscorea sp. (ñ. negro)	-27.30	2.80			root	Modern	-23.30	2.80	Keegan and DeNiro (1988)
Xantosoma sp.	-26.10	4.30			root	Modern	-22.10	4.30	Keegan and DeNiro (1988)
Xanthosoma sp.	-25.51		40.22		root	Modern	-21.51		Pestle (2010)
Xanthosoma sp.	-24.20	2.70	40.00	1.03	root	Modern	-20.20	2.70	Pestle (2010)
Xanthosoma sp.	-25.95		41.19		root	Modern	-21.95		Pestle (2010)
Xanthosoma sp.	-26.10	3.02	40.34	1.21	root	Modern	-22.10	3.02	Pestle (2010)
Xanthosoma sp.	-25.61		40.96		root	Modern	-21.61		Pestle (2010)
Xanthosoma sp.	-28.33		41.05		root	Modern	-24.33		Pestle (2010)
Xanthosoma sp.	-27.72		41.33		root	Modern	-23.72		Pestle (2010)
Xanthosoma sp.	-29.74	5.79	41.6	1.11	root	Modern	-25.74	5.79	Pestle (2010)
Manihot esculenta	-27.60	2.40			root	Modern	-23.60	2.40	Keegan and DeNiro (1988)
Tuber no identified	-24.40	3.20			root	Modern	-20.40	3.20	Keegan and DeNiro (1988)
Ipomoea batatas	-25.70	3.80			root	Modern	-21.70	3.80	Keegan and DeNiro (1988)
Ipomoea batatas	-24.78	1.73	39.79	1.01	root	Modern	-23.28		Pestle (2010)
Ipomoea batatas	-25.17		44.2		root	Modern	-21.17		Pestle (2010)
Manihot esculenta	-26.60		41.22		root	Modern	-22.60		Pestle (2010)
Manihot esculenta	-25.94		41.65		root	Modern	-21.94		Pestle (2010)

Manihot esculenta	-27.09		41.26		root	Modern	-23.09		Pestle (2010)
Manihot esculenta	-25.85		44.2		root	Modern	-21.85		Pestle (2010)
Manihot esculenta	-27.45	2.95			edible portion	Modern	-23.45	2.95	Stokes (1998)
Manihot esculenta	-27.18	6.05			edible portion	Modern	-23.18	6.05	Stokes (1998)
Manihot esculenta	-27.11	1.86			edible portion	Modern	-23.11	1.86	Stokes (1998)
Manihot esculenta	-25.99	2.95			edible portion	Modern	-21.99	2.95	Stokes (1998)
Manihot esculenta	-25.89	3.14			edible portion	Modern	-21.89	3.14	Stokes (1998)
Mean (SD)	-26.42 (1.22)	3.84 (1.56)	41.36 (1.34)	1.09 (0.09)			-22.45 (1.21)	3.84 (1.56)	
Min/Max	-29.74/-24.20	1.86/7.60	39.79/44.20	1.01/1.21			-25.74/-20.20	1.86/7.60	
**Fabaceae** (N=8)									
Fabaceae	-26.44	2.62	41.69	4.08	bean	Modern	-22.44	2.62	Pestle (2010)
Fabaceae	-27.17	3.17	42.03	4.27	bean	Modern	-23.17	3.17	Pestle (2010)
Fabaceae	-28.56	4.19	41.44	3.70	bean	Modern	-24.56	4.19	Pestle (2010)
Fabaceae	-25.78	2.75	41.46	4.19	bean	Modern	-21.78	2.75	Pestle (2010)
Fabaceae	-26.27	2.46	41.20	3.79	bean	Modern	-22.27	2.46	Pestle (2010)
Fabaceae	-27.09	1.01	42.45	3.38	bean	Modern	-23.09	1.01	Pestle (2010)
Fabaceae	-26.62	1.01	42.11	3.50	bean	Modern	-22.62	1.01	Pestle (2010)
Fabaceae	-26.97	0.65	41.82	3.63	bean	Modern	-22.97	0.65	Pestle (2010)
Mean (SD)	-26.86 (0.83)	2.23 (1.23)	41.78 (0.41)	3.82 (0.33)			-22.86 (0.83)	2.23 (1.23)	
Min/Max	-28.56/-25.78	0.65/4.19	41.20/42.45	3.38/4.27			-24.56 /-21.78	0.65/4.19	
**Tropical Fruits**									
Annona muricata (Guanábana)	-26.31	2.73	45.08	2.56	Fruit	Modern	-22.31	2.73	Pestle (2010)
Annona muricata	-27.97		41.7	-	Fruit	Modern	-23.97		Pestle (2010)

Annona muricata	-28.88		40.97	0.82	Fruit	Modern	-24.88		Pestle (2010)
Annona reticulate	-28.68	1.4	53.41	2.78	Fruit	Modern	-24.68	1.4	Pestle (2010)
Annona squamosa (Anón)	-27.67		50.28	1.07	Fruit	Modern	-23.67		Pestle (2010)
Carica papaya (Fruta bomba)	-25.89		38.25	-	Fruit	Modern	-21.89		Pestle (2010)
Carica papaya	-25.89		38.27	1.11	Fruit	Modern	-21.89		Pestle (2010)
Carica papaya	-24.48		38.87	0.96	Fruit	Modern	-20.48		Pestle (2010)
Chrysobalanus icaco (hicaco)	-26.3	0.65	46.85	2.38	Fruit	Modern	-22.3	0.65	Pestle (2010)
Chrysobalanus icaco	-26.54		41.2	-	Fruit	Modern	-22.54		Pestle (2010)
Chrysobalanus icaco	-28.07		39.07	-	Fruit	Modern	-24.07		Pestle (2010)
Chrysobalanus icaco	-28.39	4.78	-	-	edible portion	Modern	-24.39	4.78	Stokes (1998)
Chrysobalanus icaco	-27.2	4.43	-	-	edible portion	Modern	-23.2	4.43	Stokes (1998)
Coccoloba uvifera (Uva caleta)	-25.67		47.43	-	Fruit	Modern	-21.67		Pestle (2010)
Coccoloba uvifera	-24.33		46.31	0.98	Fruit	Modern	-20.33		Pestle (2010)
Coccoloba uvifera	-25.67		46.06	1.17	Fruit	Modern	-21.67		Pestle (2010)
Coccoloba uvifera	-29.51		46.83	0.89	Fruit	Modern	-25.51		Pestle (2010)
Coccoloba uvifera	-23.65	2.06	-	-	edible portion	Modern	-19.65	2.06	Stokes (1998)
Coccoloba uvifera	-23.36	2.23	-	-	edible portion	Modern	-19.36	2.23	Stokes (1998)

Cucurbita pepo (Calabaza)	-26.93	2.5	39.07	2.14	gourd	Modern	-22.93	2.5	Pestle (2010)
Cucurbita pepo	-25.41	2.58	39.66	1.81	gourd	Modern	-21.41	2.58	Pestle (2010)
Cucurbita pepo	-27.41		41.54	0.85	gourd	Modern	-23.41		Pestle (2010)
Mammea Americana (Mamey)	-28.51		40.43	-	Fruit	Modern	-24.51		Pestle (2010)
Mammea Americana	-29.92		49.72	-	Fruit	Modern	-25.92		Pestle (2010)
Manilkara bidentata (nispero)	-25.97		42.09	-	Fruit	Modern	-21.97		Pestle (2010)
Manilkara bidentata	-29.6		41.02	-	Fruit	Modern	-25.6		Pestle (2010)
Manilkara bidentata	-28.44		40.58	-	Fruit	Modern	-24.44		Pestle (2010)
Manilkara bidentata	-31.7		-	-	Leaf	Modern	-27.7		von Fisher y Tieszen (1995)
Manilkara pleeana (zapote d costa)	-27.1	7.83	-	-	edible portion	Modern	-23.1	7.83	Stokes (1998)
Manilkara pleeana	-26.45	6.36	-	-	edible portion	Modern	-22.45	6.36	Stokes (1998)
Persea americana (Aguacate)	-27.59	2.3	51.72	1.32	edible portion	Modern	-23.59	2.3	Pestle (2010)
Persea americana	-29.26	4.59	56.12	1.3	edible portion	Modern	-25.26	4.59	Pestle (2010)
Pouteria sapota (Zapote)	-29.22		43.42	-	Fruit	Modern	-25.22		Pestle (2010)
Pouteria sapota	-28.48		40.48	-	Fruit	Modern	-24.48		Pestle (2010)
Psidium guajava (Guayaba)	-27.7		46.95	1.17	Fruit	Modern	-23.7		Pestle (2010)
Psidium guajava	-27.52		40.84	-	Fruit	Modern	-23.52		Pestle (2010)
Psidium guajava	-26.68	6.74	42.88	1.01	Fruit	Modern	-22.68	6.74	Pestle (2010)

Psidium guajava	-27.87		43.19	-	Fruit	Modern	-23.87		Pestle (2010)
Spondias mombin (jobo)	-24.11		40.35	1.03	Fruit	Modern	-20.11		Pestle (2010)
Spondias mombin	-23.06		40.37	1	Fruit	Modern	-19.06		Pestle (2010)
Spondias mombin	-24.61		39.39	-	Fruit	Modern	-20.61		Pestle (2010)
Spondias mombin	-27	1.87	42.19	1.06	Fruit	Modern	-23	1.87	Pestle (2010)
Spondias purpurea	-28.66		41.8	-	Fruit	Modern	-24.66		Pestle (2010)
**Mean (SD)**	-27.06 (1.92)	3.54 (2.14)	43.46 (4.50)	1.37 (0.61)			-23.06 (1.92)	3.54 (2.14)	
**Min/Max**	-31.70/-23.06	0.65/7.83	38.25/56.12	0.82/2.78			-27.70/-19.06	0.65/7.83	

[a] The carbon isotopic composition of vegetable sources were adjusted to +1 (C_4 plants) and +4 (C_3 plants) to account for the Suess Effect taking into account the results obtained by Warinner (2010) and Warinner et al. (2013). [b] Eliminated for being considered an extreme value.

For human milk, %C and %N was obtained from Romek et al. 2013: Supplementary information (Table SI1)

%N: 1.61 (0.27); %C: 49.13 (3.63) n=96
