# Supplementary material for: Isotopic reconstruction of the weaning process in the archaeological population of Canímar Abajo, Cuba: A Bayesian probability mixing model approach
Source: PLoS One. 2017 May 1;12(5):e0176065. doi: 10.1371/journal.pone.0176065 (PMC5411105; doi:10.1371/journal.pone.0176065)
Supplement: S1 File — (PDF) [file pone.0176065.s002.pdf]

Jet Propulsion Laboratory
California Institute of Technology

JPL Image Use Policy

Unless otherwise noted, images and video on JPL public web sites (public sites ending with a jpl.nasa.gov address) may be used for any purpose without prior permission, subject to the special cases noted below. Publishers who wish to have authorization may print this page and retain it for their records; JPL does not issue image permissions on an image by image basis.

By electing to download the material from this web site the user agrees:

1. that Caltech makes no representations or warranties with respect to ownership of copyrights in the images, and does not represent others who may claim to be authors or owners of copyright of any of the images, and makes no warranties as to the quality of the images. Caltech shall not be responsible for any loss or expenses resulting from the use of the images, and you release and hold Caltech harmless from all liability arising from such use.
2. to use a credit line in connection with images. Unless otherwise noted in the caption information for an image, the credit line should be "Courtesy NASA/JPL-Caltech."
3. that the endorsement of any product or service by Caltech, JPL or NASA must not be claimed or implied.

Special Cases:

* Prior written approval must be obtained to use the NASA insignia logo (the blue "meatball" insignia), the NASA logotype (the red "worm" logo) and the NASA seal. These images may not be used by persons who are not NASA employees or on products (including Web pages) that are not NASA sponsored. In addition, no image may be used to explicitly or implicitly suggest endorsement by NASA, JPL or Caltech of commercial goods or services. Requests to use NASA logos may be directed to Bert Ulrich, Public Services Division, NASA Headquarters, Code POS, Washington, DC 20546, telephone (202) 358-1713, fax (202) 358-4331, email bert.ulrich@hq.nasa.gov.

* Prior written approval must be obtained to use the JPL logo (stylized JPL letters in red or other colors). Requests to use the JPL logo may be directed to the Institutional Communications Office, email instcomm@jpl.nasa.gov.

* If an image includes an identifiable person, using the image for commercial purposes may infringe that person's right of privacy or publicity, and permission should be obtained from the person. NASA and JPL generally do not permit likenesses of current employees to appear on commercial products. For more information, consult the NASA and JPL points of contact listed above.

* JPL/Caltech contractors and vendors who wish to use JPL images in advertising or public relation materials should direct requests to the Institutional Communications Office, email instcomm@jpl.nasa.gov.

* Some image and video materials on JPL public web sites are owned by organizations other than JPL or NASA. These owners have agreed to make their images and video available for journalistic, educational and personal uses, but restrictions are placed on commercial uses. To obtain permission for commercial use, contact

the copyright owner listed in each image caption. Ownership of images and video by parties other than JPL and NASA is noted in the caption material with each image.

Site Manager: Jon Nelson
Webmasters: Tony Greicius, Martin Perez, Luis Espinoza
