# Supplementary material for: Isotopic reconstruction of the weaning process in the archaeological population of Canímar Abajo, Cuba: A Bayesian probability mixing model approach
Source: PLoS One. 2017 May 1;12(5):e0176065. doi: 10.1371/journal.pone.0176065 (PMC5411105; doi:10.1371/journal.pone.0176065)
Supplement: S2 File — (PDF) [file pone.0176065.s003.pdf]

S2 Procedure used to account for the variation between diet and bone collagen

The C_3 and C_4 plants were adjusted following the procedure used in our previous work (Chinique de Armas et al. 2015). In that paper, following the work by Hare *et al*. (1991) and Warinner (2010), and utilizing literature-mined data (Hare *et al*., 1991; Howland *et al*., 2003; Warinner 2010, Warinner and Tuross, 2010) we derived $\Delta\delta^{13}C_{diet\text{-}col}$ and $\Delta\delta^{15}N_{diet\text{-}col}$ estimates for C_3/C_4 plant mixed (pig) diets (Chinique de Armas et al. 2015: Figure 2). Figure 2 suggested the existence of a substantial difference in exclusive C_3 and C_4 $\Delta\delta^{13}C_{diet\text{-}col}$ values (4.4 and 0.6‰, respectively), but not $\Delta\delta^{15}N_{diet\text{-}col}$ values (1.91 and 1.94‰, respectively). This values were used to account for Diet-Collagen Fractionation of Root cultigens, Legumes, Maize and Tropical fruits. For terrestrial animals the C3 values were used as terrestrial animals that were available for CA population mostly have a C3 based diet.

The diet-collagen fractionation of milk was derived from the difference keratin-milk (Romek et al., 2013) and keratin-collagen (O'Connell and Hedges, 1999; O'Connell et al., 2001).
